# The expanding role of ultrasound in acute kidney injury: from B-mode to microcirculation

**DOI:** 10.1093/ckj/sfag095

**Published:** 2026-03-19

**Authors:** Mario Meola, Ilaria Petrucci, Carlo Lomonte, Mauro Dugo, Federico Nalesso

**Affiliations:** Health Sciences Institute, Sant’Anna School of Advanced Studies, Piazza Martiri della Libertà 33 Pisa, Italy; Centro Diagnostico Apuano, SS 1 Aurelia, 2, Carrara MS; Nephrology Unit, F Miulli General Hospital, SP 127 Km 4.2 - Acquaviva delle Fonti, Italy; Nephrology and Dialysis Unit, OC Mestre-Venezia, Via Paccagnella 11, Mestre, VE; Department of Medicine (DIMED) Nephrology Unit, Via Giustiniani 2, Padova, Italy

**Keywords:** acute kidney injury, contrast-enhanced ultrasound, Doppler ultrasonography, intrarenal venous Doppler, renal microcirculation, renal resistive index, renal ultrasound

## Abstract

**Background:**

Acute kidney injury (AKI) is increasingly recognized as a dynamic systemic disorder reflecting hemodynamic instability rather than a purely nephrological complication. Conventional diagnostic criteria based on serum creatinine and urine output are delayed and provide limited insight into the underlying pathophysiological mechanisms.

**Methods:**

This narrative review summarizes current evidence on the application of multiparametric renal ultrasound in AKI. We examined the role of B-mode imaging, arterial and venous Doppler techniques, microvascular Doppler, and contrast-enhanced ultrasound (CEUS) in the assessment of renal perfusion, microcirculation, and venous congestion.

**Results:**

Doppler-derived parameters, including the renal resistive index and intrarenal venous Doppler patterns, provide dynamic information on renal hemodynamics and congestion. Microvascular Doppler and CEUS enable sensitive visualization of cortical and medullary perfusion, supporting AKI phenotyping and identification of potentially reversible mechanisms. Each modality presents specific strengths, limitations, and confounding factors.

**Conclusions:**

Multiparametric renal ultrasound complements laboratory and clinical assessment by offering real-time functional evaluation of renal perfusion and venous outflow. Within a physiology-driven framework, ultrasound may improve risk stratification, guide fluid and decongestive therapy, and support monitoring of renal recovery, shifting AKI assessment from delayed biochemical diagnosis to real-time bedside functional interpretation.

## INTRODUCTION

Acute kidney injury (AKI) represents one of the most challenging clinical syndromes in contemporary medicine. Its incidence continues to rise across healthcare settings, affecting approximately 20% of hospitalized patients, up to 50%–60% of those admitted to intensive care units, and a substantial proportion of individuals undergoing major cardiac or abdominal surgery [1–3]. Despite advances in critical care and perioperative management, AKI remains strongly associated with prolonged hospitalization, increased short-term mortality, and an elevated risk of long-term chronic kidney disease (CKD) [1, 2].

AKI should no longer be interpreted as a purely nephrological complication or an isolated decline in glomerular filtration rate (GFR). Rather, it represents a dynamic and systemic disorder reflecting the hemodynamic vulnerability of modern hospitalized patients, who are frequently exposed to hypovolemia, sepsis, inflammatory stress, nephrotoxic agents, mechanical ventilation, and fluctuations in both arterial and venous pressures. In this context, renal dysfunction often emerges as an early marker of global circulatory instability rather than as a primary renal disease.

Current diagnostic criteria for AKI rely on changes in serum creatinine (sCr) and urine output. While these parameters remain essential for staging disease severity, they provide only a delayed and indirect assessment of renal dysfunction. Serum creatinine is an imperfect surrogate for GFR in acutely ill patients, as it is influenced by muscle mass, volume status, drug exposure, and altered creatinine generation. Moreover, neither sCr nor urine output reliably reflects the underlying renal hemodynamic and microcirculatory alterations that drive injury progression or recovery [1–3].

Renal ultrasound has traditionally been used as a morphological imaging tool to assess kidney size, parenchymal echogenicity, and urinary tract obstruction. However, advances in Doppler and microvascular imaging have progressively transformed ultrasound into a dynamic bedside modality capable of interrogating renal perfusion, venous congestion, and microcirculatory function [4–9]. This transition from a static anatomical examination to a dynamic functional assessment has repositioned ultrasound as a central tool in the evaluation of AKI.

The integration of Doppler-derived indices, contrast-enhanced ultrasound (CEUS), microvascular Doppler techniques, and venous congestion assessment now allows clinicians to observe AKI as a pathophysiological process unfolding in real time rather than as a retrospective biochemical diagnosis. In this review, we synthesize experimental data, clinical evidence, and practical experience to examine how multiparametric renal ultrasound can refine AKI phenotyping, identify potentially reversible mechanisms, guide hemodynamic and fluid management, and monitor renal recovery in hospitalized and critically ill patients.

## RENAL MICROVASCULAR PHYSIOLOGY: WHY THE KIDNEY IS VULNERABLE

**Figure 1:**
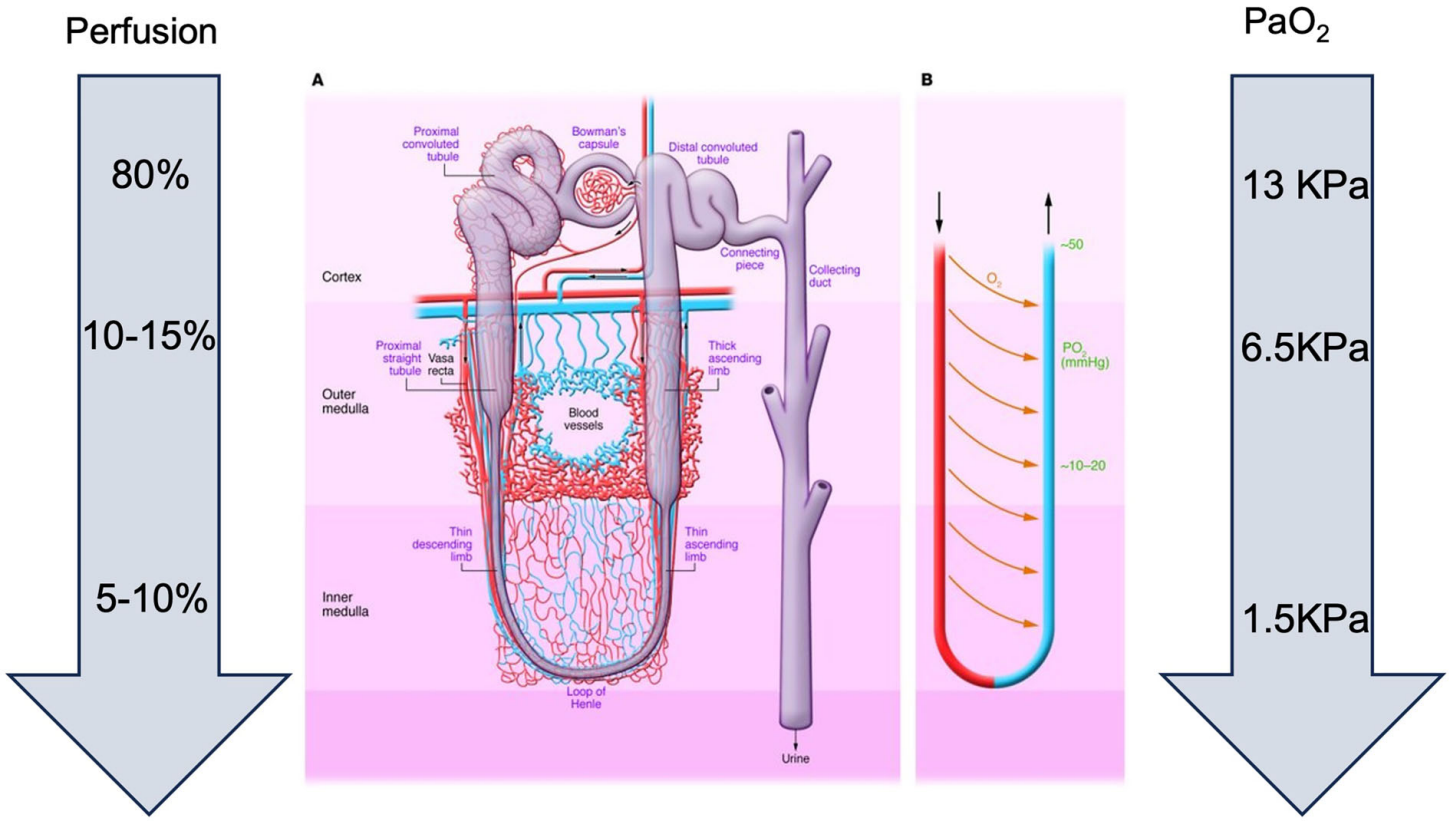
Corticomedullary oxygen gradient and microvascular vulnerability of the kidney. Schematic representation of renal microvascular organization and corticomedullary oxygen distribution. Approximately 80% of renal blood flow is directed to the cortex. In comparison, only 20% perfuses the medulla, generating a steep oxygen partial pressure gradient from ∼13 kPa in the cortex to ∼1.5 kPa in the inner medulla. Blood leaving the glomerulus supplies the peritubular capillaries and vasa recta, which sustain tubular metabolism and the countercurrent exchange system. In acute kidney injury, the preglomerular circulation and the outer medulla are particularly susceptible to vasoconstriction, reduced flow, and microvascular injury, thereby promoting medullary hypoxia.

The kidney receives approximately 20%–25% of cardiac output; however, its unique microvascular organization renders it particularly vulnerable to hypoperfusion and oxygen imbalance. Nearly 80% of renal blood flow is distributed to the cortex, whereas the outer and inner medulla operate under conditions of relative physiological hypoxia. This creates a steep corticomedullary oxygen gradient, with partial oxygen pressure decreasing from approximately 13 kPa in the cortex to nearly 1.5 kPa in the inner medulla (Fig. 1) [1–3, 10].

This asymmetric perfusion reflects the kidney’s dual functional demands. Glomerular filtration occurs at high pressure and flow in the cortex, while tubular solute transport—especially within the proximal tubule and thick ascending limb—requires substantial energy expenditure in regions characterized by limited oxygen delivery. As a result, the medulla operates close to hypoxic thresholds even under normal physiological conditions.

After leaving the glomerulus, blood from the efferent arteriole enters a portal-like microvascular network composed of peritubular capillaries and the vasa recta. This system sustains tubular epithelial metabolism and preserves the countercurrent exchange mechanism essential for urine concentration. Even modest alterations in preglomerular tone, medullary perfusion, or venous outflow may therefore precipitate a critical mismatch between oxygen delivery and metabolic demand, predisposing the kidney to ischemic injury [10, 11].

Two vascular compartments are particularly susceptible during the early phases of AKI. First, the preglomerular circulation, which is highly responsive to neurohumoral stimuli and tubuloglomerular feedback, often undergoes vasoconstriction in response to systemic hypotension, inflammation, or sympathetic activation. Second, the outer medulla—supplied by the slow-flow vasa recta—is especially vulnerable to endothelial activation, leukocyte adhesion, and microvascular obstruction, processes that further amplify regional hypoxia and tubular stress [10, 11].

This distinctive vascular architecture explains why structural abnormalities in AKI are frequently subtle or absent on conventional B-mode ultrasound, particularly in the early phases of injury. In contrast, functional alterations in arterial inflow, microvascular perfusion, and venous outflow are more readily detected using Doppler-based and CEUS techniques. These modalities provide a physiological window into the mechanisms underlying AKI before overt morphological changes or delayed biochemical abnormalities become apparent.

## PATHOPHYSIOLOGY OF AKI: FROM VASOCONSTRICTION TO TUBULAR DISRUPTION

Regardless of the initial trigger—hypovolemia, sepsis, nephrotoxic exposure, obstruction, or systemic congestion—the earliest and most unifying event in AKI is a reduction in effective renal perfusion, predominantly driven by preglomerular and medullary vasoconstriction [10, 11]. This hemodynamic response represents an adaptive attempt to preserve systemic arterial pressure but comes at the cost of reduced oxygen and nutrient delivery to metabolically active tubular segments.

The ensuing ischemia initiates a cascade of cellular and structural alterations within the tubular epithelium. Cytoskeletal disruption, characterized by actin filament depolymerization, leads to loss of epithelial polarity and opening of tight junctions. Membrane transporters, including Na⁺/K⁺-ATPase and integrins, become mislocalized to the apical membrane, impairing sodium reabsorption, and cellular adhesion. These changes promote cellular swelling, detachment from the basement membrane, and the formation of intratubular casts, which further obstruct tubular flow and increase intraluminal pressure [10].

**Figure 2:**
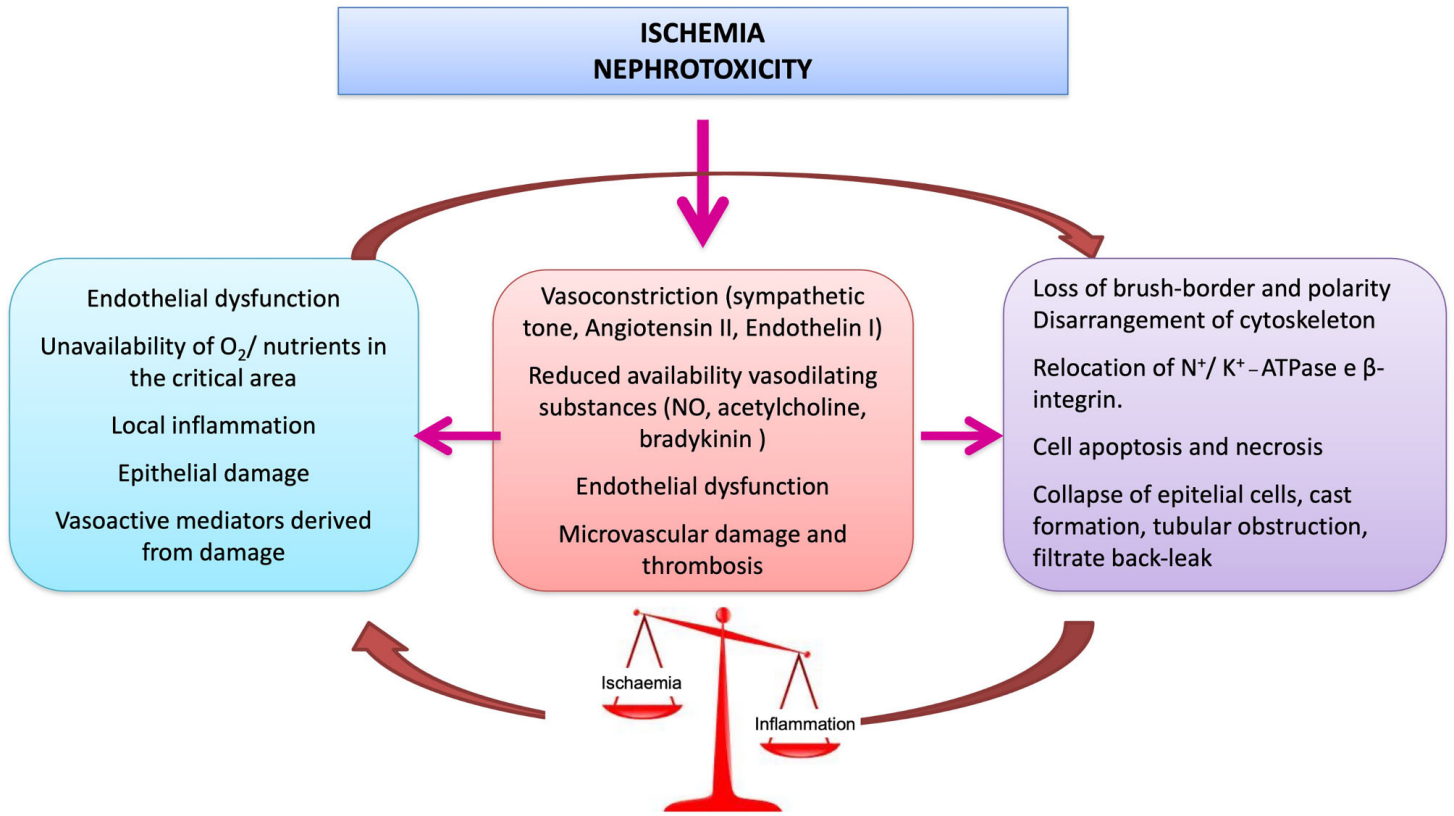
Pathophysiological model of acute kidney injury. Regardless of the primary insult, early AKI is characterized by marked renal vasoconstriction and reduced responsiveness to vasodilatory stimuli, thereby impairing oxygen and nutrient delivery to tubular cells. The resulting tubular injury is further amplified by inflammation, endothelial dysfunction, and microvascular damage, creating a self-perpetuating cycle of ischemia and tissue injury.

Concomitantly, endothelial dysfunction and inflammatory activation contribute to microvascular injury. Leukocyte adhesion, capillary rarefaction, and microthrombus formation impair peritubular and medullary blood flow, amplifying regional hypoxia. The loss of the corticomedullary osmotic gradient and backleak of filtrate into the interstitium further reduces effective glomerular filtration. Together, these mechanisms establish a self-perpetuating cycle in which tubular injury exacerbates microcirculatory dysfunction, and vice versa (Fig. 2).

Importantly, in a substantial proportion of patients, these pathophysiological processes remain potentially reversible, particularly when perfusion is restored early and sustained hypoxia is avoided. Functional recovery is primarily driven by reestablishment of microvascular flow, epithelial repair, and resolution of interstitial edema, processes that often precede normalization of sCr and urine output. Doppler-based ultrasound techniques and CEUS are uniquely suited to capture these dynamic changes in renal hemodynamics and microcirculation, providing early evidence of both injury progression and recovery [1–3].

## CLINICAL REFRAMING OF AKI: FROM “ACUTE RENAL FAILURE” TO A DISEASE SPECTRUM

Over the last two decades, international consensus groups, including KDIGO and ADQI, have progressively reframed AKI as a disease continuum rather than a binary condition defined by the need for renal replacement therapy. AKI is currently diagnosed by an acute reduction in GFR, operationally defined by an increase in sCr (≥0.3 mg/dl within 48 hours or ≥1.5 times baseline within 7 days) and/or the development of oliguria (<0.5 ml/kg/h for more than 6 hours). This conceptual shift reflects the recognition that clinically relevant kidney injury often precedes severe dysfunction [1–3, 10, 11].

Despite their widespread adoption, sCr and urine output remain late and indirect markers of renal injury. Serum creatinine rises only after a substantial decline in glomerular filtration—often exceeding 50%—and is strongly influenced by nonrenal factors such as muscle mass, volume status, drug exposure, and altered creatinine generation. Urine output, while sensitive, lacks specificity and may be affected by diuretic therapy, neurohumoral activation, and hemodynamic interventions. Importantly, neither parameter provides reliable insight into the underlying mechanism of AKI or its likelihood of reversibility [1, 2].

The limitations of functional markers have fueled extensive research into novel biomarkers of kidney injury, including cystatin C, neutrophil gelatinase-associated lipocalin, kidney injury molecule-1, and other tubular stress markers [10, 11]. While these biomarkers may detect injury earlier than sCr and offer prognostic information in selected settings, none have demonstrated sufficient accuracy to consistently discriminate among prerenal hypoperfusion, intrinsic parenchymal injury, venous congestion, or obstructive uropathy, nor to reliably guide individualized therapeutic decisions.

Increasing evidence suggests that the most clinically effective approach to AKI assessment is to integrate laboratory data, clinical evaluation, and imaging-based functional assessment. In this context, multiparametric renal ultrasound provides real-time information on renal perfusion, vascular impedance, and venous congestion, complementing biochemical markers and enhancing pathophysiological interpretation at the bedside [4, 5]. Rather than competing with biomarkers, ultrasound adds a mechanistic dimension to AKI evaluation, enabling clinicians to identify potentially reversible conditions earlier, refine risk stratification, and tailor hemodynamic and fluid management strategies.

This integrative perspective moves AKI assessment beyond retrospective biochemical diagnosis toward a dynamic, physiology-driven framework in which renal dysfunction is interpreted as the expression of evolving circulatory and microvascular alterations.

## EPIDEMIOLOGY: AKI AS A MARKER OF SYSTEMIC HEMODYNAMIC FRAGILITY

Epidemiological studies consistently demonstrate that AKI is one of the most frequent and clinically relevant complications encountered in hospitalized patients. Large observational cohorts and meta-analyses indicate that AKI develops in approximately 20%–22% of all hospitalized individuals, with incidence rates rising to 40%–60% among patients admitted to intensive care units [12, 13]. In surgical settings, AKI occurs in nearly 30%–40% of patients undergoing cardiac surgery and in 15%–25% of those undergoing major abdominal or vascular procedures [12].

The distribution of AKI etiologies further highlights its systemic nature. A substantial proportion of episodes—approximately half in most series—are attributable to prerenal or ischemic mechanisms related to hypovolemia, sepsis, low cardiac output, or systemic vasodilation. Drug-induced nephrotoxicity accounts for up to one-third of cases, reflecting the increasing pharmacological burden of contemporary hospitalized patients. Obstructive causes represent a smaller but clinically critical fraction, accounting for ∼5%–10% of AKI episodes, given their high potential for reversibility when promptly recognized [12, 13].

Importantly, epidemiological data suggest that a significant proportion of AKI events are potentially reversible, particularly when hemodynamic disturbances, venous congestion, or urinary tract obstruction are identified and corrected early. Conversely, delayed recognition and persistent injury are associated with incomplete recovery, progression to CKD, and increased long-term mortality [12, 13].

The rising incidence of AKI should not be interpreted solely as a reflection of improved detection or expanded diagnostic criteria. Rather, it mirrors the growing hemodynamic fragility of an aging and comorbid patient population increasingly exposed to complex surgical procedures, invasive ventilation, vasoactive drugs, and aggressive fluid strategies. In this context, AKI emerges as a sentinel marker of systemic circulatory stress rather than an isolated renal event.

By directly visualizing renal perfusion, vascular impedance, venous congestion, and urinary tract obstruction, ultrasound is uniquely positioned to identify reversible contributors to AKI within this vulnerable population. When integrated into routine clinical assessment, multiparametric renal ultrasound has the potential to shift AKI management from delayed reaction to early, mechanism-oriented intervention.

## B-MODE ULTRASOUND IN AKI: STRENGTHS AND DIAGNOSTIC LIMITS

Conventional B-mode ultrasound remains the first-line imaging modality for evaluating patients with AKI. It is widely available, noninvasive, repeatable, and free of ionizing radiation, making it ideally suited for bedside assessment. In the context of AKI, B-mode imaging plays a fundamental role in identifying structural abnormalities and excluding postrenal causes of renal dysfunction [14, 15].

**Figure 3:**
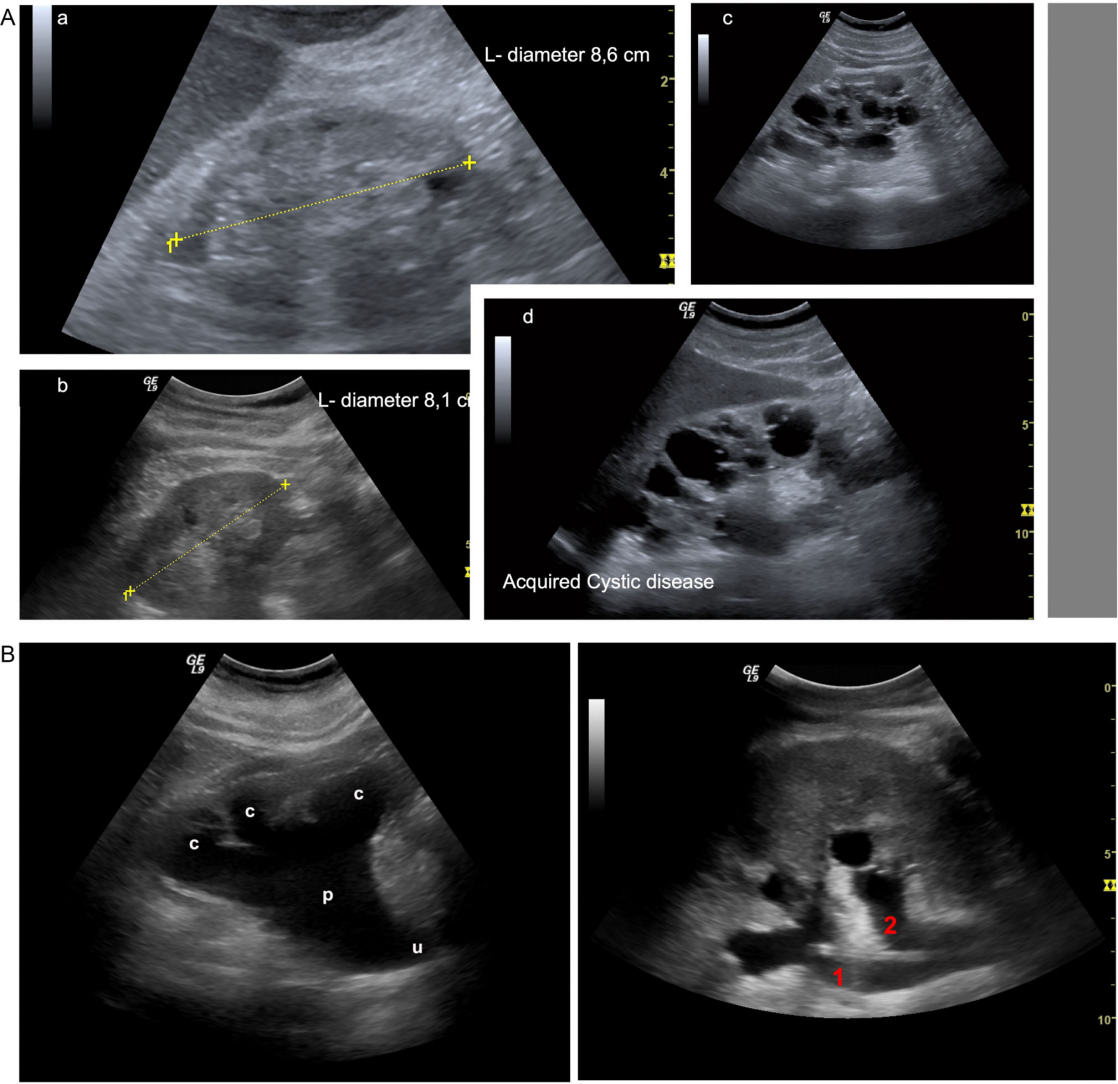
(A) Conventional B-mode ultrasound. Differential diagnosis between AKI and CKD is founded on morphologic parameters, including renal length, cortical thickness, parenchymal echogenicity, corticomedullary differentiation, the appearance of the central echogenic complex, and urinary tract status. (a, b) The kidneys in CKD are typically small (<9 cm in length), fibrotic, and hyperechoic; in advanced stages (c, d), they exhibit acquired cystic disease. (B) Obstructive nephropathy. Ultrasonography demonstrates high sensitivity in diagnosing urine stasis. When the upper urinary tract is dilated, the renal sinus appears occupied by an anechoic, multiloculated fluid collection resembling a carnation flower. The purpose of B-mode ultrasound in this context is to identify, when possible, the luminal, parietal, or external cause of obstruction. (a) Severe and persistent hydronephrosis with ectasia of the calyces, pelvis, and ureter. (b) Presents a case of hydronephrosis involving a double collecting system.

Key morphological parameters assessed on B-mode ultrasound include renal length, cortical thickness, parenchymal echogenicity, corticomedullary differentiation, and status of the collecting system. In adults, normal renal length typically ranges from 10.5 to 11.5 cm, with a parenchymal thickness exceeding 13–14 mm. Although these measurements are not diagnostic in isolation, they provide essential contextual information. Reduced kidney size, cortical thinning, increased echogenicity, and loss of corticomedullary differentiation are characteristic of CKD, whereas kidney size is usually preserved or mildly increased in acute processes (Fig. 3A).

B-mode ultrasound is indispensable for the rapid detection of urinary tract obstruction, which accounts for ∼5%–10% of AKI cases and is among the most readily reversible causes when promptly treated. Hydronephrosis, dilatation of the renal pelvis and calyces, and, in selected cases, visualization of obstructive lesions allow immediate diagnostic orientation and therapeutic intervention (Fig. 3B). In this setting, B-mode imaging is both sensitive and clinically decisive.

**Figure 4:**
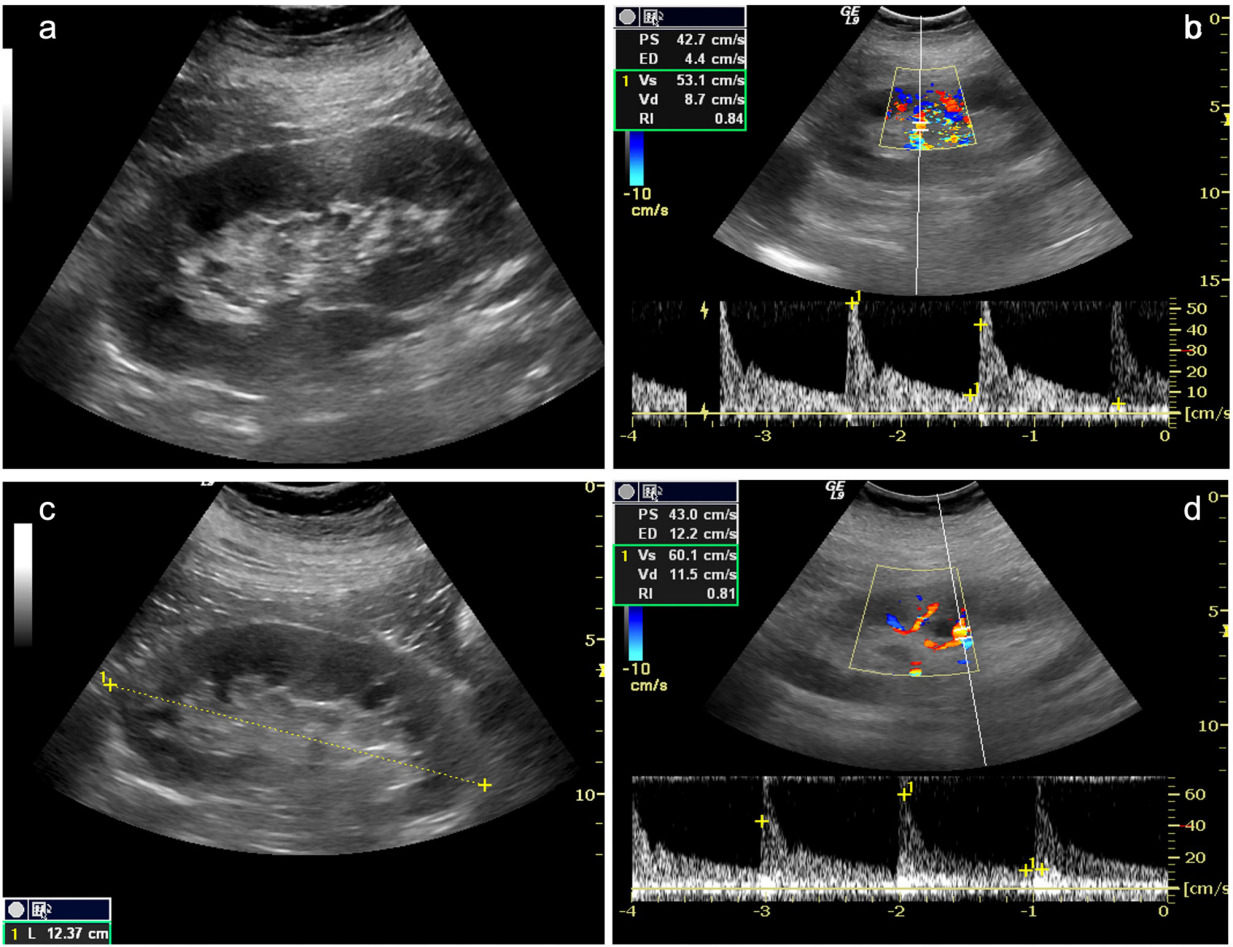
Acute kidney injury secondary to favism. Acute hemolysis following fava bean ingestion leads to massive intravascular hemoglobin release, oxidative stress. Free hemoglobin and heme pigments contribute to tubular toxicity, intrarenal vasoconstriction, and microcirculatory dysfunction, resulting in pigment-induced acute kidney injury. (a, c) B-mode ultrasound shows bilaterally enlarged kidneys (>12 cm) with diffusely hypoechoic echotexture, without specific alterations in corticomedullary differentiation. (b, d) The GFR impairment, as indicated by a sudden increase in sCr and oliguria, is explained by markedly elevated RRI (>0.80) in both kidneys, reflecting severe intrarenal vasoconstriction and tubular damage.

In contrast, in intrinsic forms of AKI—particularly ischemic or toxic acute tubular necrosis—B-mode findings are frequently subtle or nonspecific (Fig. 4). The kidneys may appear mildly enlarged, with hypoechoic parenchyma and swollen medullary pyramids, while corticomedullary differentiation is often preserved. These features lack sufficient sensitivity and specificity to reliably characterize the underlying mechanism of injury or to predict reversibility. As a result, B-mode imaging alone cannot adequately discriminate among prerenal hypoperfusion, intrinsic parenchymal damage, and hemodynamically mediated AKI.

**Figure 5:**
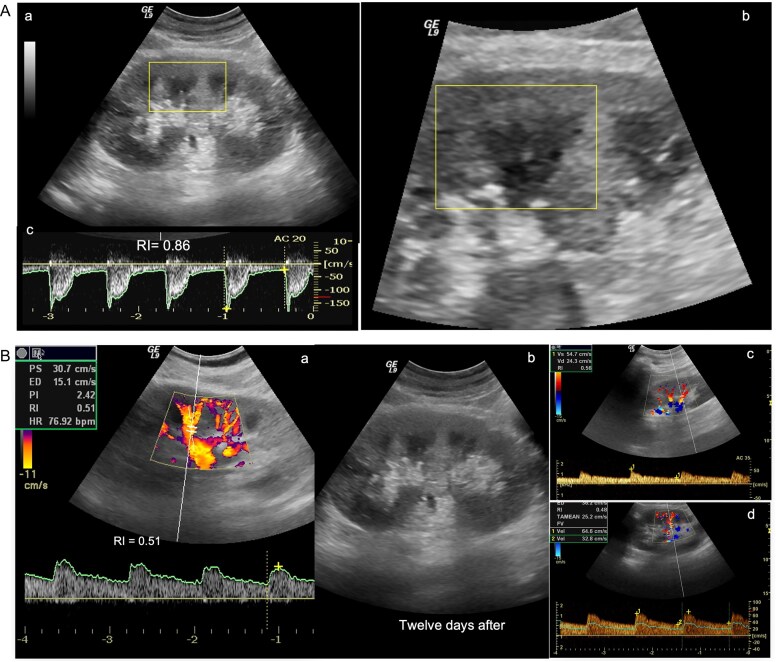
(A) Acute kidney injury secondary to antifungal toxicity (acute phase). Following exposure to an antifungal agent, the patient presented with oliguria and increased serum creatinine levels (2.9 mg/dl). (a) B-mode ultrasound shows bilaterally enlarged, globose kidneys with diffusely hypoechoic parenchyma and (b) edematous, swollen medullary pyramids (magnified area), without specific alterations of corticomedullary differentiation. These morphologic findings are nonspecific and cannot reliably distinguish the underlying nature of parenchymal injury. (c) Spectral Doppler demonstrates a marked increase in intrarenal resistive index (RI > 0.80), consistent with severe intrarenal vasoconstriction and supporting a rapid diagnosis of toxic acute tubular necrosis. (B) Functional recovery after antifungal-induced acute kidney injury. Twelve days after the acute insult, during the polyuric recovery phase, intrarenal resistive index normalizes (RI < 0.60) despite persistent morphologic abnormalities on B-mode ultrasound. The early decrease in RI precedes normalization of serum creatinine and reflects recovery of renal microcirculation, highlighting RI as a dynamic functional marker of renal recovery.

This diagnostic limitation underscores the inherent limitation of purely morphological assessment in a condition characterized by functional and microcirculatory alterations. While B-mode ultrasound is essential for excluding obstruction, contextualizing renal size and chronicity, and guiding further imaging, it provides only a partial view of renal pathophysiology in AKI. Functional assessment using Doppler-based techniques is therefore required to interrogate renal perfusion, vascular impedance, and hemodynamic stress, bridging the gap between morphology and renal function (Fig. 5A, B).

## DOPPLER TECHNIQUES AND RENAL PERFUSION

Doppler-based ultrasound techniques extend renal imaging beyond morphology by providing real-time information on blood flow and tissue perfusion. In AKI, these modalities offer a functional complement to laboratory markers, enabling direct visualization of hemodynamic alterations that underlie changes in glomerular filtration [5, 16].

### Color Doppler, power Doppler, and microvascular Doppler

**Figure 6:**
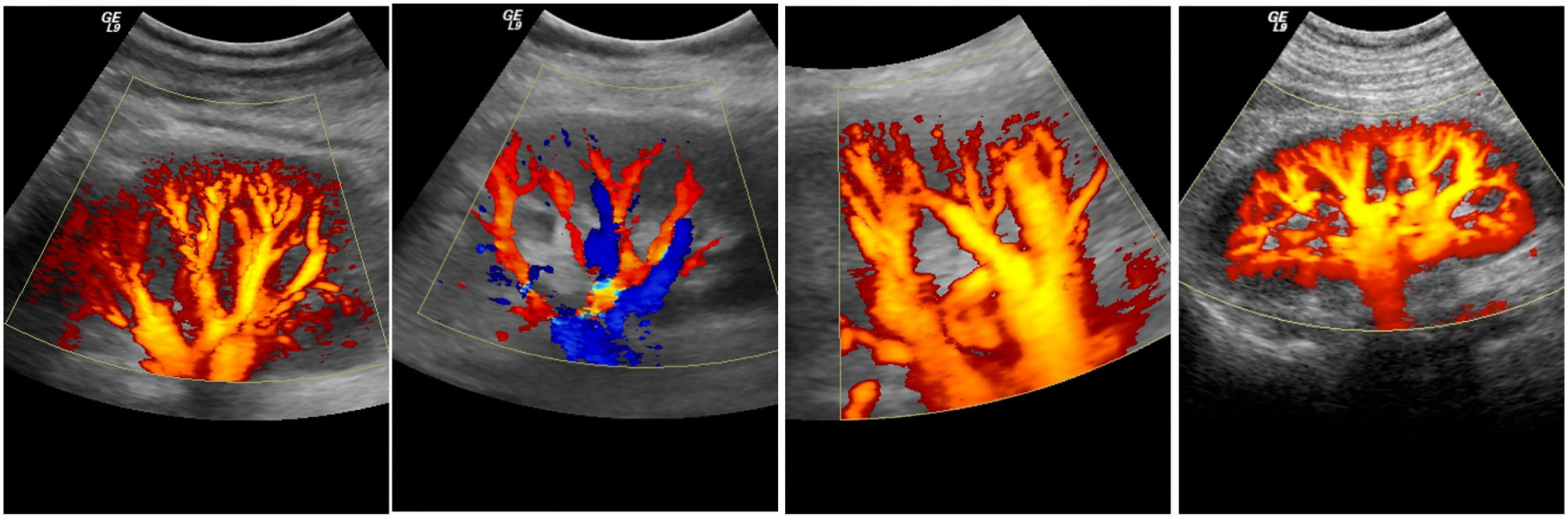
Color-power Doppler. CD-PD Doppler, as well as microvascular imaging, allow a rapid, global evaluation of renal perfusion. Absence or marked reduction of signal within the main renal artery, its segmental branches or cortical blushing raises suspicion for vascular occlusion or severe hypoperfusion.

Color Doppler ultrasound allows a rapid, global assessment of renal perfusion. Visualization of flow within the main renal artery and its segmental branches confirms macroscopic arterial patency, whereas the absence or marked reduction of the Doppler signal raises suspicion for severe hypoperfusion, arterial thrombosis, or technical limitations related to low flow states. However, color Doppler is relatively insensitive to slow-flow conditions and may underestimate residual perfusion in vasoconstricted or hypotensive patients. Power Doppler improves sensitivity to low-velocity flow by displaying the amplitude of Doppler signals rather than flow direction. This feature makes it particularly useful for assessing cortical perfusion in states of reduced renal blood flow, such as shock or severe vasoconstriction, where conventional color Doppler may fail to detect intraparenchymal vessels (Fig. 6).

More recently, microvascular Doppler techniques, including superb microvascular imaging (SMI), have further enhanced the ability to visualize very low–velocity flow within the cortical and medullary microcirculation [16]. By selectively suppressing motion artifacts while preserving slow-flow signals, SMI enables visualization of peritubular and medullary vessels without the need for contrast agents. In patients with AKI, SMI often demonstrates patchy or globally reduced cortical perfusion, providing an early visual marker of microcirculatory distress [5, 6].

Despite their clinical utility, color Doppler, power Doppler, and microvascular Doppler techniques remain predominantly qualitative. Collectively, they address a fundamental yet limited question: whether the kidney is perfused and, if so, how perfusion is spatially distributed. While valuable for detecting gross perfusion abnormalities, these techniques do not quantify the hemodynamic forces governing intrarenal blood flow or their temporal dynamics. Such information requires spectral Doppler analysis.

### Spectral Doppler and renal resistive index

Spectral Doppler assessment of intrarenal arteries provides quantitative information on intrarenal hemodynamics by measuring peak systolic velocity, end-diastolic velocity, and derived indices such as the renal resistive index (RI). The renal RI should therefore not be interpreted as a direct measure of intrarenal vascular resistance. Rather, it represents a composite hemodynamic signal that reflects the complex interplay among arterial pulsatility, vascular compliance, interstitial pressure, and downstream venous impedance [17–19].

In the setting of AKI, RI commonly increases in response to preglomerular vasoconstriction, medullary hypoxia, tubular edema, and elevated interstitial pressure. Values exceeding 0.75–0.80 have been reported across ischemic, septic, toxic, and congestive forms of AKI and have been associated with greater severity, progression, and increased likelihood of renal replacement therapy [14, 20]. Importantly, RI behaves as a dynamic hemodynamic biomarker rather than a static diagnostic threshold.

Experimental and clinical studies indicate that RI often rises earlier than sCr during acute injury and decreases earlier during recovery, reflecting restoration of microcirculatory flow before biochemical normalization [20, 21]. Serial RI measurements, therefore, provide more clinically meaningful information than isolated values, particularly when interpreted in relation to hemodynamic interventions, fluid management, and vasoactive therapy.

In obstructive AKI, RI exhibits characteristic lateralization, with significantly higher values in the affected kidney compared with the contralateral side. Following relief of obstruction, RI typically declines rapidly—often preceding structural normalization—supporting its role as an early marker of functional recovery [22].

Interpretation of RI requires careful consideration of confounding factors [23]. Systemic arterial stiffness, heart rate, mechanical ventilation, positive end-expiratory pressure, intra-abdominal pressure, and vasoactive medications can significantly influence intrarenal Doppler waveforms. Consequently, RI should not be used in isolation to diagnose AKI or to infer specific mechanisms. When integrated into a multiparametric ultrasound assessment, however, RI contributes to AKI phenotyping, risk stratification, and monitoring of renal response to therapeutic interventions [23–29]. Table 1 summarizes the principal physiological determinants and clinical interpretation of RI behavior in AKI.

**Table 1:** Hemodynamic interpretation of renal resistive index behavior in acute kidney injury.

AKI phenotype	Predominant mechanism	Typical RI behavior	Spatial distribution	Temporal dynamics	Clinical interpretation
Prerenal (functional hypoperfusion)	Reversible preglomerular vasoconstriction	Mild–moderate increase (≈0.70–0.75)	Bilateral, symmetric	Rapid normalization after volume or flow restoration	Adaptive response; not indicative of structural injury
Congestive/cardiorenal AKI	Elevated renal venous pressure, reduced microvascular compliance	Moderate–marked increase (>0.75)	Bilateral	Decreases in parallel with decongestion	Marker of venous congestion rather than ischemia
Septic/shock-associated AKI	Macro–microcirculatory uncoupling, shunting, endothelial dysfunction	Early and marked increase (>0.77–0.80)	Bilateral	Rises before serum creatinine; normalizes early during recovery	Dynamic prognostic marker; associated with severity and need for RRT
Ischemic or toxic intrinsic AKI	Tubular edema, interstitial pressure elevation, microvascular injury	Persistent elevation (>0.80)	Bilateral	Slow or incomplete normalization	Reflects severe microcirculatory and interstitial damage
Acute interstitial nephritis	Diffuse interstitial inflammation and edema	Disproportionate increase	Bilateral	Variable; may persist despite systemic improvement	Indicates interstitial compartment involvement
Unilateral obstructive AKI	Increased tubular and interstitial pressure distal to obstruction	Marked elevation on affected side	Lateralized	Rapid decline after relief of obstruction	Functional and highly sensitive marker of obstruction
Bilateral obstructive AKI	Bilateral downstream pressure overload	Bilateral elevation	Symmetric	Depends on duration and reversibility	May mimic intrinsic AKI without lateralization

**Table 2:** Intrarenal venous Doppler patterns and therapeutic implications in AKI.

Venous Doppler pattern	Hemodynamic interpretation	Typical clinical context	Therapeutic implications
Continuous venous flow	Normal or mildly elevated RVP	Euvolemia, early AKI, arterial hypoperfusion	Fluids may be considered if hypoperfusion is suspected
Mildly pulsatile flow	Early venous congestion	Fluid loading, early right heart dysfunction	Caution with fluids; reassess volume strategy
Biphasic venous flow	Significant venous congestion	Right-sided or biventricular failure, elevated CVP	Initiate or intensify decongestive therapy (diuretics, UF)
Monophasic/discontinuous flow	Severe renal venous hypertension (“renal tamponade”)	Advanced heart failure, severe CRS, postoperative congestion	Avoid fluids; aggressive decongestion, reassess ventilation and CVP
High-grade VExUS	Systemic venous congestion	Cardiac surgery, ICU AKI, CRS	Guide decongestive strategy and monitor response over time

## CONTRAST-ENHANCED ULTRASOUND (CEUS): DIRECT ASSESSMENT OF RENAL MICROCIRCULATION

CEUS represents the most sensitive bedside ultrasound technique for evaluating renal perfusion and microcirculation. The use of microbubble contrast agents, which remain strictly intravascular, allows real-time visualization of cortical and medullary blood flow with high temporal resolution, without exposing patients to ionizing radiation or nephrotoxic contrast media. These characteristics make CEUS particularly attractive in patients with AKI, in whom iodinated or gadolinium-based contrast agents are often contraindicated.

In AKI, CEUS provides functional information that cannot be obtained with conventional Doppler techniques [30–34]. Alterations in cortical enhancement, delayed or heterogeneous medullary perfusion, and segmental perfusion defects may be detected before overt changes in sCr occur, enabling earlier identification of ischemic or toxic tubular injury. CEUS is especially valuable in differentiating global hypoperfusion from focal vascular insults, such as renal artery thrombosis, cholesterol embolization, or segmental ischemia, which may be missed or underestimated by standard Doppler imaging. The potential of CEUS for evaluating renal perfusion is particularly evident in renal transplantation, where the superficial location of the graft provides excellent acoustic windows and detailed visualization of cortical microvascularization. CEUS may rapidly confirm the absence of parenchymal perfusion in graft vascular complications, such as acute renal artery thrombosis or segmental ischemia. The accompanying video clips illustrate the role of CEUS in identifying segmental ischemia of the renal graft (Movies 1 and 2–Supplementary file).

In postoperative and transplant settings, CEUS has demonstrated utility in distinguishing delayed graft function, acute rejection, vascular complications, and drug-related injury based on characteristic enhancement patterns. Serial CEUS examinations may also support monitoring of renal reperfusion following shock resolution, decongestive therapy, or relief of urinary tract obstruction, offering insight into microvascular recovery that precedes biochemical improvement.

Despite its high sensitivity for detecting perfusion abnormalities, CEUS should not be considered a screening tool for all patients with AKI. Its optimal role is problem-oriented, reserved for cases in which conventional B-mode and Doppler findings are inconclusive or when detailed assessment of renal microcirculation is likely to influence clinical management. Furthermore, CEUS requires operator expertise and strict adherence to standardized acquisition protocols to ensure reproducibility [30–34].

A major limitation of CEUS in AKI remains the lack of robust, standardized, and reproducible quantitative perfusion parameters. Current clinical applications rely largely on qualitative or semiquantitative interpretation of enhancement patterns and time–intensity curves, which are influenced by acquisition settings, region-of-interest selection, and operator experience [28–32]. As a result, CEUS does not yet provide a reliable measure of absolute or relative changes in renal microcirculatory perfusion over time, limiting its use for precise longitudinal monitoring or inter-patient comparison [30–34].

Future developments in renal CEUS will depend on the validation of standardized acquisition protocols, the identification of reliable quantitative perfusion metrics, and integration with advanced postprocessing techniques, including artificial intelligence–based analysis. These advances may allow CEUS to evolve from a predominantly qualitative imaging modality into a quantitative tool for functional assessment of renal microcirculation in AKI.

## VENOUS CONGESTION AND AKI: A FUNCTIONAL AND THERAPEUTIC DRIVER

AKI has traditionally been interpreted primarily as a consequence of impaired arterial inflow and reduced renal perfusion pressure. Increasing experimental and clinical evidence, however, indicates that venous congestion and elevated renal venous pressure play an equally critical role in limiting glomerular filtration, particularly in contemporary hospitalized and critically ill patients [35–41].

Elevation of central venous pressure increases renal interstitial and intratubular pressure, thereby reducing the transglomerular filtration gradient even in the presence of preserved or near-normal arterial inflow. This mechanism is especially relevant in conditions characterized by right-sided or biventricular cardiac dysfunction, pulmonary hypertension, volume overload, and positive pressure ventilation. In these settings, AKI may develop despite apparently adequate cardiac output and mean arterial pressure, highlighting the limitations of arterial-centric interpretations of renal dysfunction.

From a hemodynamic perspective, two predominant patterns of AKI can be identified. In states of predominantly arterial hypoperfusion, typically associated with low cardiac output or systemic vasodilation, renal dysfunction is characterized by high intrarenal arterial resistance, reduced diastolic flow, and globally impaired renal perfusion. In contrast, in predominantly venous congestive states, renal arterial inflow may be relatively preserved, while venous drainage is severely impaired. Chronic elevation of renal venous pressure leads to interstitial edema, increased intrarenal pressure, and a functional form of “renal tamponade,” ultimately reducing effective glomerular filtration.

**Figure 7:**
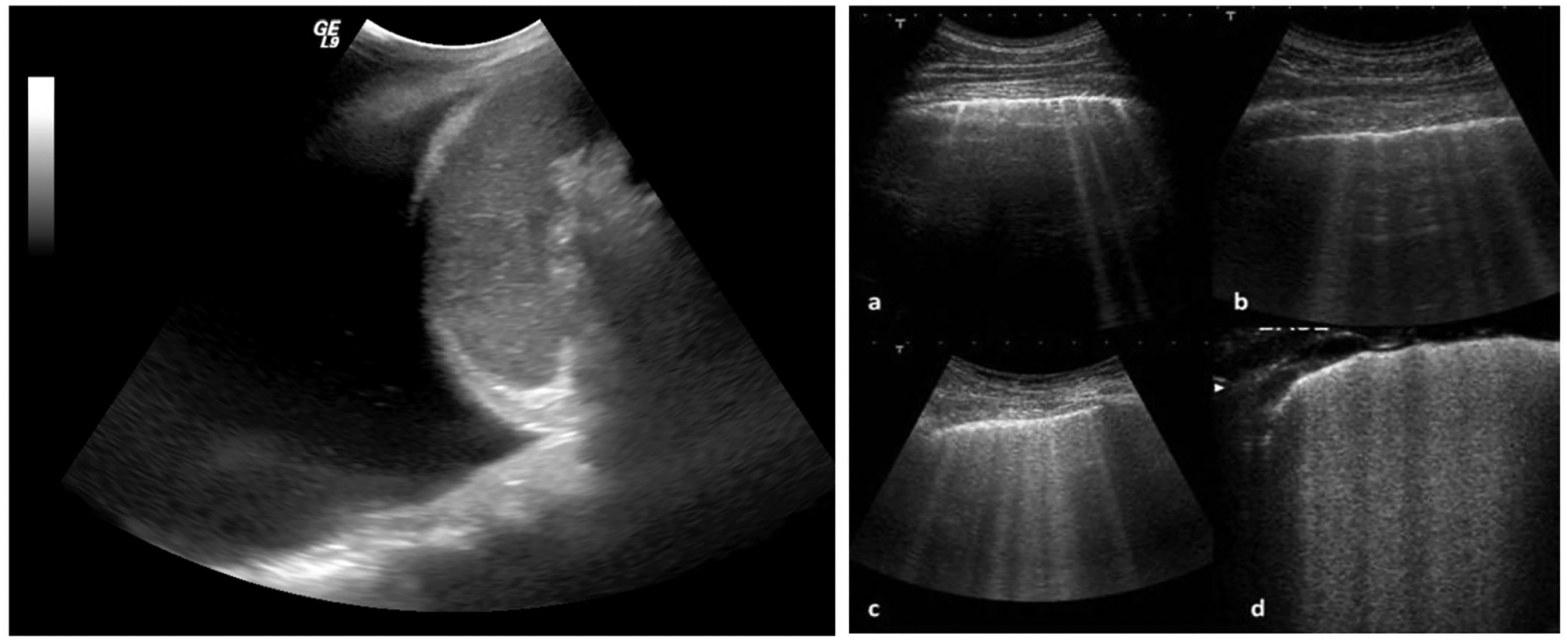
Acute left-sided heart failure. In this condition, reduced cardiac output leads to systemic vasoconstriction. Renal, musculoskeletal, and splanchnic perfusion decreases to preserve vital organs. Renal Doppler ultrasound shows high-resistance intrarenal arterial waveform with reduced or absent diastolic flow, indicating low–cardiac output. Due to the patient’s critical condition, assessing RI variability from respiratory changes is often not feasible. Transthoracic echocardiography may show a left-sided pleural effusion, a thin pericardial effusion, and pulmonary B-lines suggestive of interstitial edema.

Intrarenal venous Doppler provides a direct, real-time assessment of renal outflow impedance and represents the final common pathway of venous congestion affecting the kidney. As renal venous pressure increases, venous Doppler waveforms evolve from continuous to pulsatile, biphasic, and eventually monophasic patterns, reflecting progressive transmission of right atrial pressure into the renal parenchyma. Loss or reversal of the systolic (S) wave and marked pulsatility have been associated with elevated central venous pressure (often exceeding 15–20 mmHg) and with worsening renal function and adverse clinical outcomes (Fig. 7) [37–39].

Unlike indirect surrogates of congestion, such as inferior vena cava diameter or collapsibility alone, intrarenal venous Doppler directly reflects the hemodynamic burden imposed on the kidney. For this reason, venous Doppler abnormalities carry important therapeutic implications. In the presence of marked venous congestion, further fluid administration may exacerbate renal dysfunction, whereas a decongestive strategy—based on diuretics, ultrafiltration, or ventilatory optimization—may lead to functional improvement.

**Figure 8:**
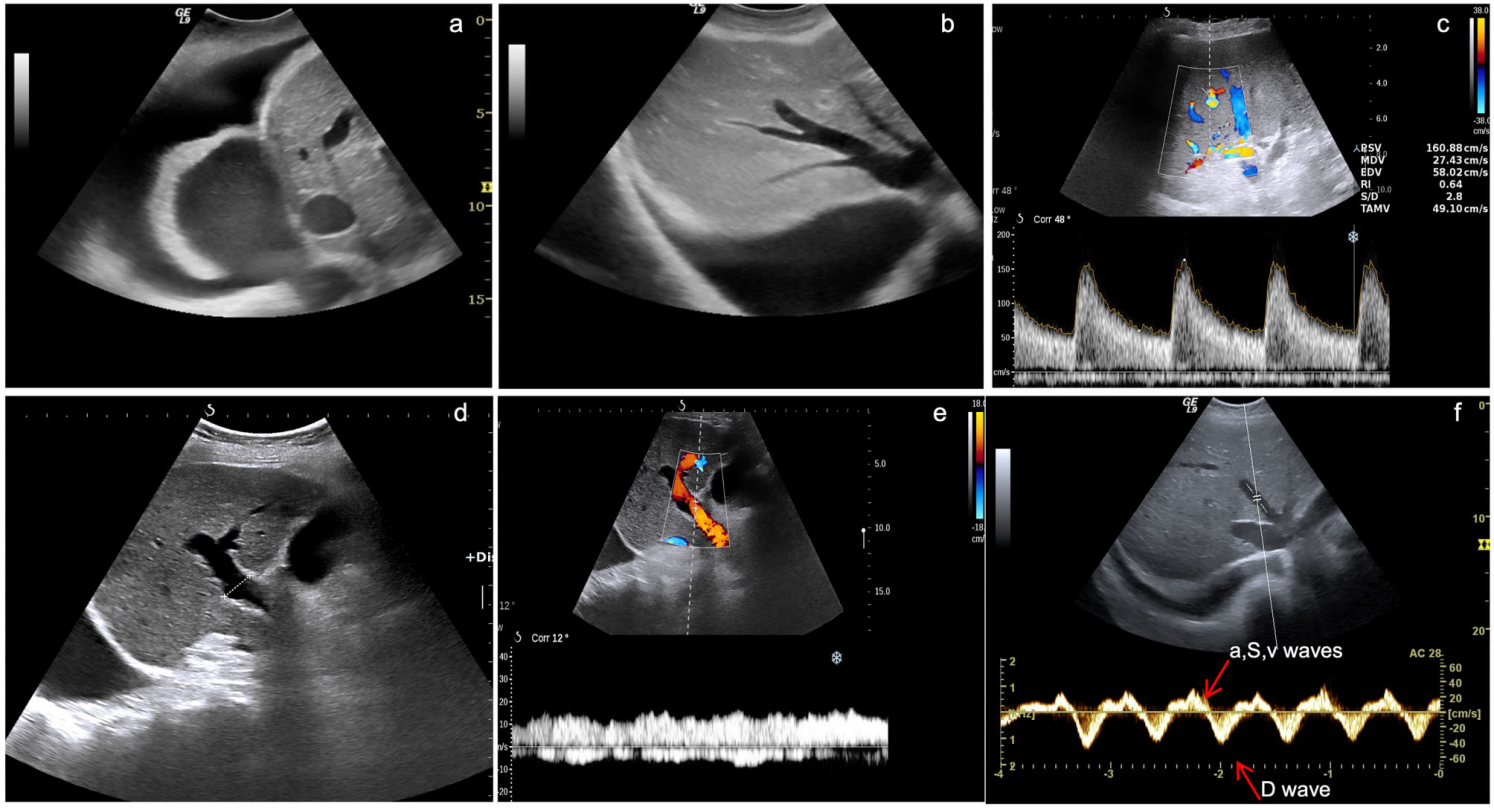
Systemic venous congestion in type 2 cardiorenal syndrome. Chronic CVP elevation in heart failure causes systemic volume overload affecting serous cavities, IVC, liver, hepatic veins, splenoportal axis, intestines, and kidneys. (a) Coronal lateral and (b) subcostal ultrasound show right pleural effusion with exudative features. (a, b, d, e) The liver is congested, with dilated confluence of the hepatic veins. The portal vein has no significant dilation and flow velocity <10 cm/s. (c) The spleen is enlarged and congested (intrasplenic RI > 0.83). (f) The hepatic vein waveform exhibits spectral changes indicative of right-sided pressure overload. The case involves a patient with chronic heart failure and severe tricuspid regurgitation, with an inverted S/D ratio; the S wave fuses with the a and v waves.

The Venous Excess Ultrasound (VExUS) score integrates intrarenal venous Doppler with Doppler assessment of the hepatic veins, portal vein, and inferior vena cava to provide a semi-quantitative estimate of systemic venous congestion. High-grade VExUS has been consistently associated with the development, persistence, and severity of AKI, particularly in patients undergoing cardiac surgery and in critically ill populations [35, 36, 40, 41]. Although current evidence is largely observational, VExUS offers a structured and reproducible framework for congestion assessment at the bedside (Fig. 8).

**Figure 9:**
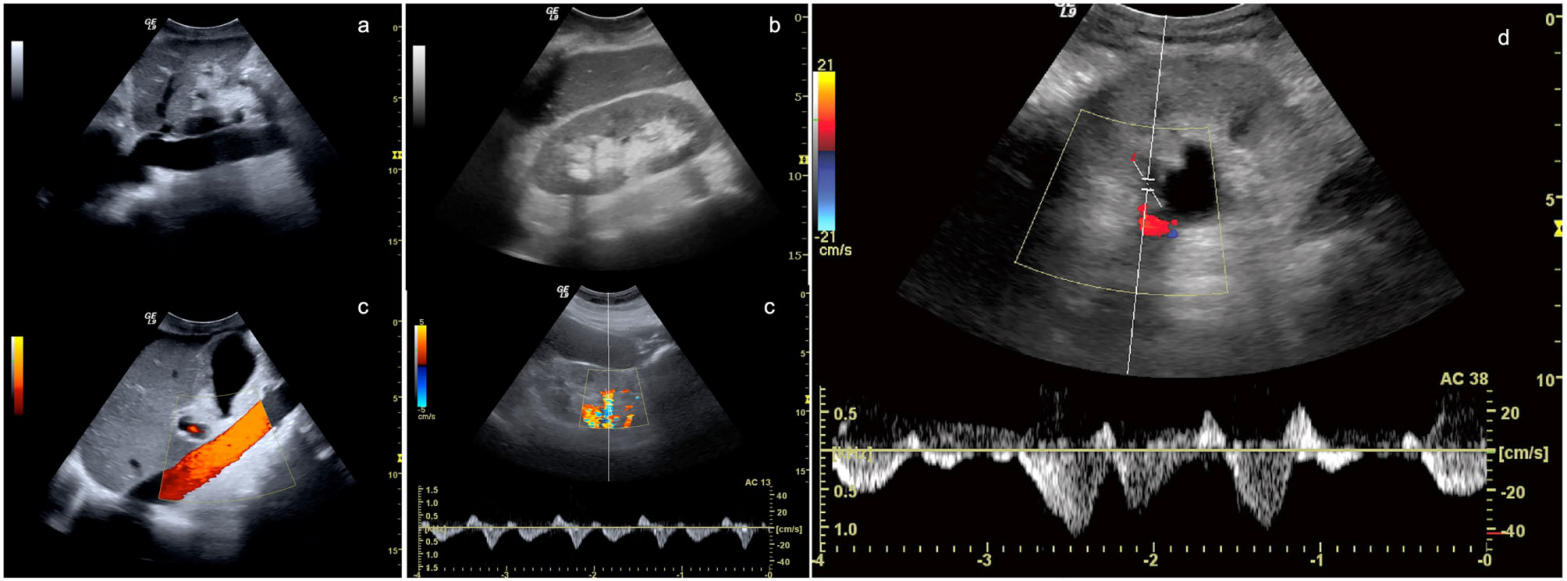
Ultrasound markers of systemic venous congestion. (a) The B-mode and power Doppler sagittal views of the IVC show a dilated vessel (>2.5 cm diameter, collapsibility <50%), indicating elevated central venous pressure (>15–20 cmH₂O) and right-sided volume overload. The kidney appears enlarged and congested; intrarenal venous waveforms become increasingly pulsatile and multiphasic, resembling hepatic flow. Venous flow progress from continuous to pulsatile, biphasic, then discontinuous and monophasic as congestion worsens. In severe renal venous hypertension, seen in advanced heart failure, cardiorenal syndrome, and postoperative congestion, ultrasound findings support a decongestive approach—reducing fluids and using diuretics, ultrafiltration, or ventilatory management.

Importantly, venous congestion represents a modifiable contributor to AKI. Serial evaluation of intrarenal venous Doppler patterns and VExUS grading allows clinicians to monitor renal response to decongestive interventions and tailor therapy dynamically. In this physiology-driven approach, renal ultrasound evolves from a purely diagnostic tool into a real-time monitor of treatment response, enabling timely identification and correction of reversible congestive mechanisms of AKI (Fig. 9).

## OBSTRUCTIVE AKI: PATHOPHYSIOLOGY AND ULTRASOUND PATTERNS

Obstructive nephropathy accounts for ∼5%–10% of AKI cases but represents one of the most readily reversible forms when promptly recognized and treated [42]. In this context, renal ultrasound plays a pivotal diagnostic role by combining morphological assessment with functional hemodynamic evaluation.

The pathophysiological response to acute urinary tract obstruction evolves dynamically over time. In the very early phase, typically within the first hours after obstruction, prostaglandin-mediated vasodilation may transiently increase renal blood flow, occasionally resulting in a paradoxical reduction of the renal resistive index. This phase is short-lived and often clinically silent. With sustained obstruction beyond 6–12 hours, progressive elevation of intrapelvic pressure leads to tubular hypertension, compression of peritubular capillaries, and activation of intrarenal vasoconstrictive mechanisms. These changes result in reduced medullary perfusion, tubular epithelial injury, and a marked increase in intrarenal vascular impedance.

On B-mode ultrasound, hydronephrosis remains the hallmark of obstructive AKI, manifesting as dilatation of the renal pelvis and calyces. However, the degree of pelvicalyceal dilatation does not reliably reflect the severity or functional impact of obstruction. Early obstruction may occur in the absence of significant hydronephrosis, whereas chronic obstruction may show marked dilatation with relatively preserved renal function. This limitation underscores the importance of adjunct functional assessment.

Spectral Doppler interrogation of intrarenal arteries provides critical functional information in obstructive AKI. A characteristic feature is the lateralization of the RRI, with significantly higher values in the obstructed kidney compared with the contralateral side. RI values frequently exceed 0.85 in the affected kidney, whereas the nonobstructed kidney typically maintains values within or near the normal range. This asymmetry enhances diagnostic confidence, particularly in equivocal or early cases.

Following relief of obstruction, a rapid hemodynamic rebound is commonly observed, often accompanied by a polyuric phase. Importantly, the RRI typically decreases promptly—often within hours to days—prior to normalization of renal morphology and sCr. This early decline in RI reflects the restoration of medullary perfusion and a reduction in intrarenal pressure, underscoring the value of Doppler ultrasound in monitoring functional recovery.

Taken together, these observations demonstrate that dynamic changes in intrarenal Doppler indices provide greater diagnostic and prognostic insight than static anatomical grading of hydronephrosis alone. In obstructive AKI, renal ultrasound thus serves not only as a diagnostic tool but also as a functional monitor of reversibility and therapeutic response.

## ELASTOGRAPHY: PROMISE, LIMITATIONS, AND FUTURE DIRECTIONS

Ultrasound elastography has recently emerged as a potential noninvasive tool for assessing renal mechanical properties and investigating tissue alterations in patients with AKI [43]. Shear wave elastography (SWE) allows quantitative assessment of tissue stiffness by measuring the propagation velocity of mechanically induced shear waves within the renal parenchyma [44].

Preliminary clinical studies suggest that renal stiffness values may be increased in patients with AKI compared with non-AKI critically ill patients. In a recent prospective study including 103 critically ill patients, SWE measurements demonstrated significantly higher stiffness values in several renal segments in patients with AKI, with moderate diagnostic performance for AKI detection (AUC values ranging from ∼0.73–0.78 depending on the renal region analyzed). Optimal diagnostic cut-off values were reported around 9.9 kPa in the upper pole medulla and ∼2.9–4.4 kPa in cortical and medullary regions [45].

However, interpretation of renal elastography remains complex. Unlike hepatic elastography—where stiffness primarily reflects fibrosis—renal stiffness is influenced by multiple dynamic physiological factors. Renal perfusion pressure, interstitial edema, tubular injury, and systemic or intrarenal venous congestion may all significantly modify stiffness measurements. These hemodynamic determinants are particularly relevant in critically ill patients, in whom rapid changes in renal blood flow and venous pressure frequently occur.

In addition, the kidney presents intrinsic anatomical and biomechanical challenges for elastography, including marked cortical–medullary heterogeneity and tissue anisotropy. Experimental and clinical observations demonstrate substantial variability in stiffness measurements depending on the measurement plane and renal segment analyzed, which may further limit reproducibility [45, 46].

Consequently, although renal elastography represents a promising research tool for exploring biomechanical changes in AKI and may potentially contribute to early detection or prognostic stratification, current evidence remains limited and heterogeneous. At present, elastography should therefore be considered an adjunctive investigational technique rather than a routine component of ultrasound assessment in patients with AKI.

## MULTIPARAMETRIC RENAL ULTRASOUND: A PRACTICAL BEDSIDE WORKFLOW

No single ultrasound parameter can fully characterize the complexity of AKI; however, when integrated into a structured multiparametric approach, ultrasound provides complementary information that enhances diagnostic accuracy and guides clinical management.

A stepwise bedside workflow may be adopted to systematically evaluate patients with AKI. Initial B-mode imaging should focus on kidney size, parenchymal echogenicity, corticomedullary differentiation, and urinary tract anatomy, with particular attention to excluding obstructive causes. This step establishes the anatomical context and differentiates acute processes from chronic structural disease.

Subsequently, Doppler assessment of intrarenal arteries allows evaluation of arterial inflow and vascular impedance. Measurement of the RRI, preferably performed serially and in conjunction with clinical and hemodynamic data, provides insight into microvascular stress, interstitial pressure, and dynamic response to therapeutic interventions. Comparison between kidneys is particularly informative in suspected obstructive or asymmetric disease.

In patients with suspected or confirmed systemic congestion, intrarenal venous Doppler should be incorporated early into the assessment. Identification of pulsatile, biphasic, or monophasic venous waveforms indicates impaired renal outflow and supports a decongestive rather than volume-expanding strategy. When available, integration of intrarenal venous Doppler into the VExUS score provides a structured framework for grading congestion severity and monitoring response to therapy.

CEUS and microvascular Doppler techniques should be reserved for problem-oriented evaluation. These modalities are particularly useful when conventional imaging fails to explain the severity or persistence of AKI, when focal perfusion defects are suspected, or when detailed assessment of cortical and medullary microcirculation may influence clinical decision-making.

Finally, interpretation of multiparametric renal ultrasound requires awareness of potential confounders, including systemic hemodynamics, mechanical ventilation, vasoactive drugs, intra-abdominal pressure, and pre-existing vascular disease. Ultrasound findings should therefore be integrated with laboratory data and clinical assessment rather than interpreted in isolation.

By following a structured and physiology-driven workflow, multiparametric renal ultrasound becomes a practical bedside tool capable of identifying reversible mechanisms, guiding fluid and hemodynamic management, assessing AKI severity, and monitoring renal recovery over time. Table 3 summarizes the physiological meaning and clinical implications of multiparametric renal ultrasound parameters across the main AKI phenotypes.

**Table 3:** Multiparametric renal ultrasound in AKI: physiological meaning and clinical implications.

Ultrasound modality	Main parameter	Physiological information	Clinical implications in AKI
B-mode ultrasound	Kidney size, cortical thickness, echogenicity	Structural integrity, chronicity, edema	Differentiation between AKI and CKD; detection of obstruction
Color Doppler	Presence and distribution of flow	Global renal perfusion	Identification of severe hypoperfusion or vascular occlusion
Power Doppler	Cortical perfusion signal	Low-velocity flow sensitivity	Assessment of residual perfusion in hypotension or shock
Microvascular Doppler (SMI)	Cortical and medullary microflow	Microcirculatory integrity	Early detection of microvascular distress
Spectral Doppler	Renal resistive index (RI)	Intrarenal vascular impedance and compliance	AKI severity, prognosis, monitoring of recovery

**Figure 10:**
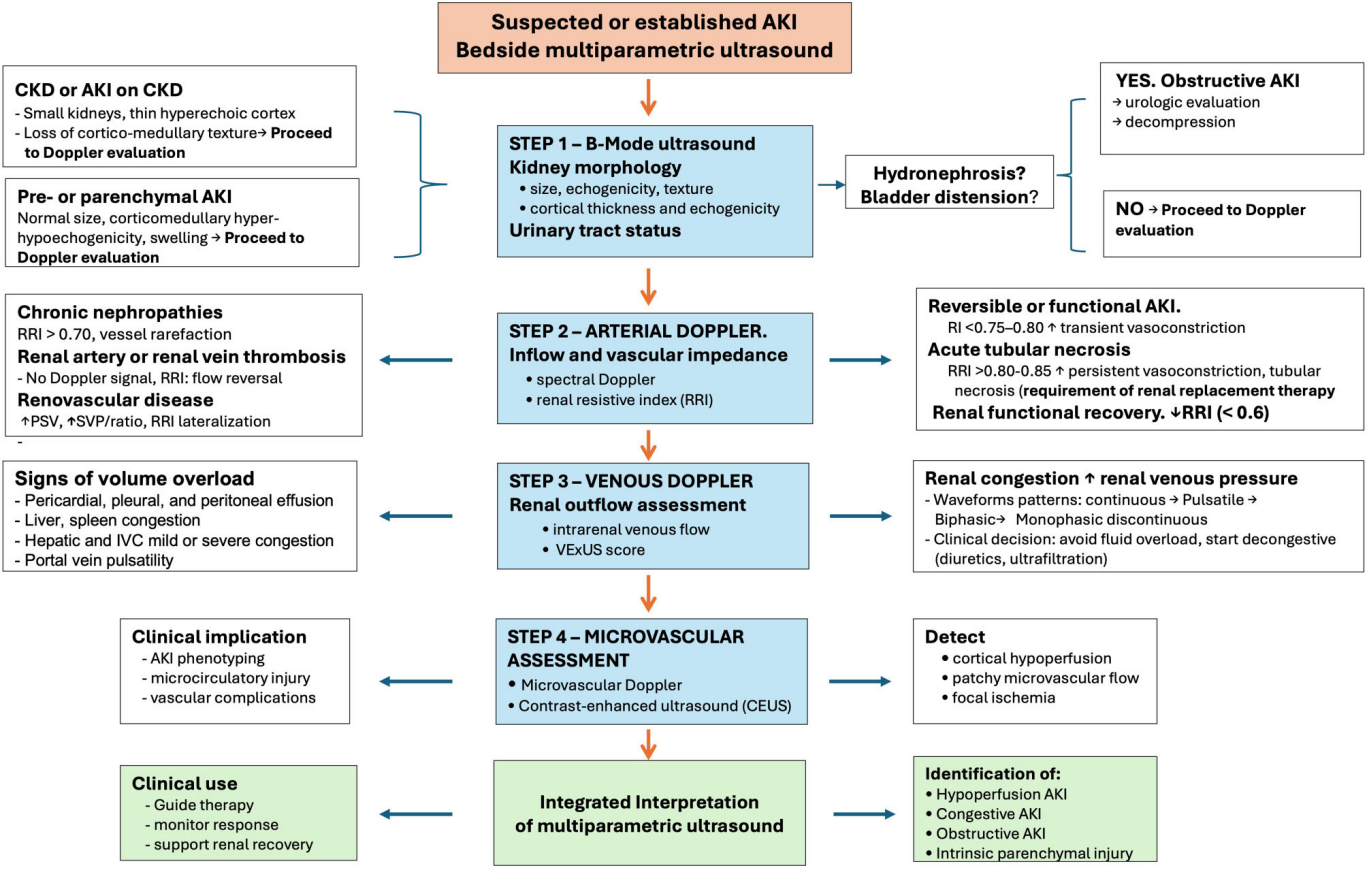
Multiparametric ultrasound algorithm for the bedside evaluation of acute kidney injury. A stepwise multiparametric ultrasound approach integrates B-mode imaging, arterial Doppler, venous Doppler, and microvascular techniques to characterize the hemodynamic mechanisms underlying acute kidney injury. Initial B-mode examination focuses on renal morphology and exclusion of obstructive causes. Spectral Doppler assessment of intrarenal arteries provides information on renal vascular impedance and microvascular stress through analysis of the renal resistive index. Intrarenal venous Doppler and VExUS evaluation allow identification of renal venous congestion and guide decongestive strategies. When conventional ultrasound findings are inconclusive, microvascular Doppler and contrast-enhanced ultrasound enable direct visualization of renal microcirculation and perfusion abnormalities. The integration of these modalities supports a physiology-based interpretation of AKI and facilitates mechanism-oriented bedside management.

A stepwise bedside workflow may be adopted to systematically evaluate patients with AKI (Fig. 10). The proposed workflow emphasizes integrating morphological, arterial, venous, and microvascular ultrasound signals to support mechanism-oriented AKI phenotyping and targeted clinical management.

## LIMITATIONS OF MULTIPARAMETRIC ULTRASOUND IN AKI

The interpretation of multiparametric renal ultrasound in AKI requires awareness of several methodological and practical limitations. Doppler-based parameters are inherently operator-dependent and may be influenced by technical factors such as insonation angle, patient positioning, and acoustic window quality. Inter-observer variability may also affect the reproducibility of measurements, particularly when evaluating intrarenal venous waveforms or subtle variations in Doppler indices.

In addition, several systemic factors can significantly modify intrarenal Doppler patterns independently of intrinsic renal injury. Mechanical ventilation, positive end-expiratory pressure, intra-abdominal pressure, heart rate variability, and vasoactive medications may alter renal arterial and venous waveforms, potentially confounding interpretation.

The acquisition and interpretation of advanced techniques, including CEUS and microvascular Doppler imaging, also require specific training and standardized protocols. Furthermore, the availability of these modalities remains heterogeneous across institutions and clinical settings.

For these reasons, multiparametric ultrasound findings should not be interpreted in isolation but rather integrated with clinical evaluation, laboratory data, and systemic hemodynamic assessment. When used within this physiological framework, ultrasound provides valuable functional insight into the mechanisms underlying AKI while acknowledging its intrinsic methodological limitations.

## CONCLUSIONS

AKI should no longer be interpreted solely as a biochemical abnormality defined by delayed changes in sCr and urine output. Rather, it represents a dynamic disorder driven by alterations in renal perfusion, microcirculation, and venous outflow, which often precede and outlast measurable changes in conventional laboratory markers.

Multiparametric renal ultrasound uniquely captures this complexity at the bedside. B-mode imaging remains essential for identifying structural abnormalities and excluding obstructive causes of AKI, while Doppler-based techniques provide functional insight into arterial inflow, intrarenal vascular impedance, and microcirculatory stress. The RRI provides dynamic information on the evolution of injury and recovery when interpreted within the appropriate hemodynamic context. Intrarenal venous Doppler and venous congestion scoring highlight the often under-recognized role of renal venous hypertension and support targeted decongestive strategies. CEUS further complements these modalities by enabling sensitive visualization of cortical and medullary microcirculation, although standardized quantitative assessment remains limited.

When integrated into a physiology-driven framework, renal ultrasound evolves from a purely diagnostic tool into a real-time functional monitor capable of identifying potentially reversible mechanisms, guiding hemodynamic and fluid management, refining risk stratification, and monitoring renal recovery. This integrated approach bridges nephrology and systemic hemodynamics, repositioning the kidney as an active participant in critical illness rather than a passive victim of systemic dysfunction.

Future research should focus on standardizing acquisition protocols, validating quantitative perfusion metrics, and conducting prospective studies evaluating ultrasound-guided management strategies. As these gaps are addressed, multiparametric renal ultrasound evolves from a purely diagnostic tool into a real-time functional monitor in the clinical assessment and management of AKI.

## Supplementary Material

sfag095_Supplemental_File

## Data Availability

The data underlying this article will be shared on reasonable request to the corresponding author.
