# Supplementary material for: The expanding role of ultrasound in acute kidney injury: from B-mode to microcirculation
Source: Clin Kidney J. 2026 Mar 19;19(7):sfag095. doi: 10.1093/ckj/sfag095 (PMC13339950; doi:10.1093/ckj/sfag095)
Supplement: sfag095_Supplemental_File [file sfag095_supplemental_file.docx]

**Supplementary file**

**Comment Movie 1. Contrast-enhanced ultrasound (CEUS) in renal transplantation.** The indications for contrast-enhanced ultrasound (CEUS) of the native kidney, as described in the EFSUMB guidelines, are largely applicable to renal transplantation, although controlled and randomized studies in transplant recipients remain lacking. Evidence from case reports and small clinical series suggests that CEUS is a valuable imaging modality for assessing renal perfusion, particularly given the graft’s superficial location and the favorable acoustic window.

The primary clinical indications include the evaluation of ischemic necrosis due to thrombosis of the main renal artery or vein, as well as cortical necrosis related to hyperacute rejection or thromboembolic phenomena. In the present case, the observed necrotic areas are related to thrombosis of an accessory arterial branch supplying a segment of the graft.

**Comment Movie 2. Contrast-enhanced ultrasound (CEUS) in renal transplantation.** In acute renal artery thrombosis, CEUS represents a particularly effective imaging modality for confirming the absence of parenchymal perfusion. However, color Doppler, power Doppler, and microvascular imaging techniques may also demonstrate the absence of segmental perfusion with comparable diagnostic accuracy. In the case presented here, the graft appears completely non-perfused on CEUS examination. Interestingly, partial retrograde filling of the renal vein can still be observed.
